# Long-Term Effectiveness of Treating Dentin Hypersensitivity with Bifluorid 10 and Futurabond U: A Split-Mouth Randomized Double-Blind Clinical Trial

**DOI:** 10.3390/jcm10102085

**Published:** 2021-05-12

**Authors:** Marta Mazur, Maciej Jedliński, Artnora Ndokaj, Roman Ardan, Joanna Janiszewska-Olszowska, Gianna Maria Nardi, Livia Ottolenghi, Fabrizio Guerra

**Affiliations:** 1 Department of Dental and Maxillofacial Sciences, Sapienza University of Rome, 00161 Rome, Italy; maciej.jedlinski@pum.edu.pl (M.J.); artnora.ndokaj@uniroma1.it (A.N.); Giannamaria.nardi@uniroma1.it (G.M.N.); livia.ottolenghi@uniroma1.it (L.O.); fabrizio.guerra@uniroma1.it (F.G.); 2 Department of Interdisciplinary Dentistry, Pomeranian Medical University in Szczecin, 70-111 Szczecin, Poland; jjo@pum.edu.pl; 3 Chair of Econometrics, Department of Economic Sciences, Koszalin University of Technology, 75-343 Koszalin, Poland; roman.ardan@tu.koszalin.pl

**Keywords:** dentin hypersensitivity, fluoride varnish, bonding resign, tooth wear, pain reduction, exposed dentin

## Abstract

Background: The definition of dental hypersensitivity is “pain derived from exposed dentin in response to chemical, thermal tactile or osmotic stimuli which cannot be explained as arising from any other dental defect or disease”. One of the treatments proposed is tubular occlusion. The aim of this in vivo split-mouth randomized clinical trial was to evaluate the clinical efficacy of a in-office application of a fluoride varnish (Bifluorid 10) and a bonding resin (Futurabond U) in adults with dentin hypersensitivity. Material and methods: A total of 180 teeth were treated with Bifluorid 10 and 160 with Futurabond U. Outcome measurements were taken one or two weeks before treatment, at baseline at the application days, at 1 week and at 1–6 months after first treatment. Results: Both treatments reduced pain intensity. Bifluorid 10 and Futurabond U have similar efficacy in reducing SCHIFF-measured pain reduction, while Bifluorid 10 is significantly more efficient for VAS-measured pain reduction, mainly due to long-term pain reduction. Patient age has a significant negative influence on pain reduction, while the influence of patient gender and BEWE of the tooth is insignificant. Conclusions: Bifluorid 10 and Futurabond U are effective in the treatment of dental hypersensitivity. The RCT was registered at the US National Institutes of Health (ClinicalTrials.gov) #NCT04813848.

## 1. Introduction

The definition of dental hypersensitivity is “pain derived from exposed dentin in response to chemical, thermal tactile or osmotic stimuli which cannot be explained as arising from any other dental defect or disease” [1]. Despite the fact that it is known to be characterized by a high prevalence, there is no consensus among scientists as to its frequency. One review reports its prevalence from various studies ranging from 1% to 98% [2], whereas another [3] from 5% to 85%. This variability highlights both the scale of the problem and lack of standardization in diagnosis. In clinical practice, the diagnosis is provided only after excluding other causes of unpleasant sensations. Several clinical conditions could cause symptoms similar to dental hypersensitivity; thus, there is a need for differential diagnosis [4]. The most common symptoms arise from pulpitis caused by caries or a trauma, such as enamel/dentine fracture [5]. Moreover, dental hypersensitivity can develop as a result of enamel loss caused by erosion, abrasion, attrition and cementum loss subsequent to gingival recession [6].

No specific pathogenesis has been assigned to dental hypersensitivity from nerve fiber conduction, dentine fiber conduction, dentine tubule lymphatic conduction; however, most of the studies gave support to Brannstrom’s hydrodynamic theory [7,8,9]. In accordance with this theory, a stimulus applied to open tubules of the dentin (due to non-carious lesions) increases the flow of dentinal tubular fluid. Increased flow and higher pressure cause mechanical deformation of type Aƌ nerves from Rashkov’s weave located in the inner ends of the tubules, and is, with a high certainty, responsible for producing pain [10,11]. It has been found that tubule diameters were significantly larger in a hypersensitive area compared to a non-sensitive surface [12] and that the number of open tubules per surface area in the exposed dentin of hypersensitive teeth can be eight times larger than that of the teeth non-responsive to stimuli [12]; this further confirms this thesis.

Hypersensitivity is a problem which makes everyday existence difficult. It lowers general wellbeing, influences food choices, social life and even the patient’s overall self-esteem [13]. For this reason, patients are willing to undergo therapy to prevent pain. The patient may try to treat mild hypersensitivity at home. Domestic desensitizing treatment includes toothpastes, mouthwashes and chewing gum [14]. With higher intensity and frequency of pain, the patient should be examined by a dentist. In-office desensitizing treatment measures comprise gels, solutions, varnishes, resin sealers and dentin adhesives. Some of the most popular measures in present dental care are adhesive fluoride varnishes and self-etching adhesives. In between visits, the patient should use the previously mentioned domestic prophylaxis measures, as recommended by the dental practitioner [15].

The primary aim of the present study was to compare the effectiveness of two products for in-office use (Bifluorid 10 vs. Futurabond U—*both VOCO, Cuxhaven, Niedersachsen, Germany*) on long-term pain reduction and the secondary aim was to evaluate possible personal factors that can influence the effectiveness of the therapy.

## 2. Materials and Methods

### 2.1. Study Design and Setting

A randomized, double blind, split-mouth controlled study was conducted in patients with dentin hypersensitivity. The study was performed at the 1st Observation Unit of Department of Oral and Maxillofacial Sciences, “Sapienza” University of Rome. Consecutive healthy adults with dentine hypersensitivity were enrolled. Recruitment was carried out between September and December 2019; a six-month follow up was performed between May and October 2020.

The study was approved by the Local Ethical Committee (n. 5877) and all patients have signed an informed consent. All the procedures were in accordance with the 1964 Helsinki Declaration and its later amendments or comparable ethical standards. The experimentation followed CONSORT guideline and was registered at the US National Institutes of Health (ClinicalTrials.gov) #NCT04813848.

### 2.2. Study Population

(a) Inclusion criteria

The inclusion criteria was healthy adults, aged ≥ 18, with ≥1 sensitive tooth and with exposed dentin in the upper or lower dental arches. The presence of an equivalent contralateral tooth for comparison to the examined tooth with exposed dentin was mandatory to include a patient in this study. 

Schiff sensitivity score needed to be ≥2, indicating that application of a jet of air makes the patient respond and move or requests discontinuation of the stimuli application.

(b) Exclusion criteria
- domestic or in-office fluoride application and bleaching 6 months before beginning of treatment;
- long-term use of anti-inflammatory, analgesic and psychotropic drugs;
- allergies to product ingredients;
- eating disorders, systemic conditions that cause or predispose patients to develop dentin hypersensitivity (for example, gastroesophageal reflux disease, GERD);
- excessive dietary or environmental exposure to acids (vegan or vegetarian or diet with daily use of fruit and vegetable smoothies/extracts);
- orthodontic appliance treatment within the previous three months;
- periodontal surgery within the previous three months before the study.

### 2.3. Sample Size Calculation

Sample size calculation was performed for 2-paired means with a 1:1 allocation ratio, assuming normality, for a 2-sided test with a split-mouth design. The present study was a paired observation case, with the participant serving as control, since both intervention and comparison treatments are applied in the same patient. This design is efficient, since the sites that receive interventions are similar, thus reducing variance and sample-size requirements. The following formula was applied n = f(alpha, beta)*\sigma^2/(differenza^2)), where \sigma was the standard deviation of the within-person differences and f(α, β) was a function of power and significance level. In the present study, detecting a clinically significant difference of 0.53 units following treatment as measured on the Schiff sensitivity scale, with a standard deviation of 1.06 units, requires a sample of 30 subjects, each with two different treatment methods. Considering a dropout rate of 10%, a total of 40 subjects will be enrolled.

### 2.4. Randomized Allocation

A split-mouth design was performed: the two treatments were randomly allocated on the left and right side of the dental arches. Whenever possible, the operator selected study teeth in both the maxillary and mandibular dental arches of each subject. The allocation was done on the basis of the year of birth date: the even numbers started with the left side (the first treatment will be always the Bifluorid 10 varnish), while the odd numbers started with the application of the varnish on the right side. In both cases, the bonding resin was applied on the opposite dental arch.

### 2.5. Blinding Procedure

Blinding of patients, data collectors and outcome adjudicators were achieved.

### 2.6. Description of the Desensitizing Agents

- Two different desensitizers were used: Bifluorid 10 (VOCO GmbH) and Futurabond U (VOCO GmbH). The desensitizers were applied by one dentist (MM) experienced in conservative and preventive dentistry and in epidemiologic surveys.
- Bifluorid 10 is a single dose transparent, colophony-free varnish with 5% sodium fluoride (22.600 ppm fluoride) and 5% calcium fluoride.
- The application was performed according to the manufacturer’s instructions. The varnish was applied on a clean and dry surface, then left for 10–20 s to allow the fluoride varnish to be absorbed, then it was dried with air. Patients were instructed not to brush their teeth for the next 12–24 h. In this study, the application was repeated 3 times at intervals of 7 days according to the manufacturer’s instruction. Repeated applications should only involve drying of surfaces and reapplication of the varnish.
- Futurabond U is a single dose dual curing universal adhesive. Futurabond U application was performed according to the manufacturer’s instruction for cases with hypersensitive tooth necks. The bonding was applied without etching, on a clean and dry surface. After 10 s, any excess material was removed and then light cured for 30 s.

### 2.7. Co-Interventions and Standardization

The domestic oral care of the participants was standardized: the same toothpaste, toothbrush and interproximal cleaner were delivered to all the subjects. Patients received instructions concerning domestic oral hygiene procedures based on the tailored brushing method (TBM) [16].

### 2.8. Assessment of the Clinical Variables

Basic erosive wear examination index (BEWE), Schiff sensitivity scale and Vas scale were assessed on the vestibular surfaces of incisors, cuspids and bicuspids with accessible cervical regions.

Basic erosive wear examination (BEWE) is a tool for screening for erosive tooth wear and recording clinical findings and assisting in the decision making process as a guide for management. It is a four-level score that grades severity of erosive wear:

(0) no surface loss;
(1) initial loss of surface texture;
(2) distinct defect, hard tissue loss less than 50% of the surface area;
(3) hard tissue loss in more than 50% of the surface area.

Buccal/facial, occlusal and lingual/palatal surfaces were examined in sextants (17–14, 13–23, 24–27, 37–34, 33–43 and 44–47) and the highest score was recorded (maximum sum score = 18). From the sum score, four categories were defined; the first two categories required monitoring and general advice, whereas the two latter comprised complex strategies until the maximum approach, including restorative measures [16].

### 2.9. Level of Pain Assessment 

The Schiff sensitivity scale was used to assess dentin hypersensitivity. A score of 0, 1, 2 or 3 was assigned by the participant. A score of 0 = no subject response to stimulus; 1 = subject response but will continue; 2 = subject responds and moves or requests discontinuation; 3 = painful response to stimulus, discontinuation requested. The lower the score, the lower the hypersensitivity. Air-blast stimulus was used: the response to a jet of cold air was rated by the examiner [17].

VAS scale Magnitude estimation requires subjects to indicate the level of pain experienced along a continuum represented by a visual analog scale (VAS). At each evaluation, subjects recorded the sensitivity of each tooth to timed (5 s) applications of compressed air (from a three-way dental unit syringe at a distance of approximately 2 cm) and a cold stimulus (ice stick contacting the tooth surface) on the VAS [17]. 

### 2.10. Outcome Measurements

Primary outcome in pain/sensitivity changes was assessed by the patient on application of cold and air-blast stimuli. Magnitude estimation required subjects to indicate the level of pain experienced along a continuum represented by VAS and according to the Schiff sensitivity scale. The outcome measurements were assessed and collected by another experienced dentist with skills in conservative and preventive dentistry (AN).

### 2.11. Duration of the Study and Times of Follow-Up

The study period was 6 months. Patients were treated at baseline (T0) and at 7 days (T1), 1 month (T2), 2 months (T3) and 6 months (T4). Clinical variables were recorded at the enrollment, at baseline and at all times of follow-up.

### 2.12. Statistical Analysis

The efficacy of treatment in both groups and between-group differences was assessed using Student’s t-test. Regression models were used to estimate the effect of age and gender and BEWE of a tooth on pain reduction in both groups according to both scales. The results were considered statistically significant at *p* < 0.05. 

The R statistical package (The R Foundation for Statistical Computing, Wirtschaftsuniversität Wien, Vienna, Austria) was used for the calculations. 

## 3. Results

A total of 300 patients were screened, 50 of those fulfilled the inclusion criteria and were enrolled in the protocol. Twenty-two patients were lost at follow-up, due to problems arising from the COVID-19 pandemic.

A total of 340 teeth were treated in 28 adult patients aged 23.3–69.3 years (mean = 46.9, SD = 13.3). There were 21 female patients aged 23.3–69.3 years (mean = 48.5, SD = 12.2) and 7 male patients aged 25.2–64.1 years (mean = 42.0, SD = 16.2). The number teeth treated in a single patient varied from 6 to 18 with mean = 12.2 and SD = 4.0.

### 3.1. Comparison of Bifluorid 10 and Futurabond U Efficacy

**Figure 1:**
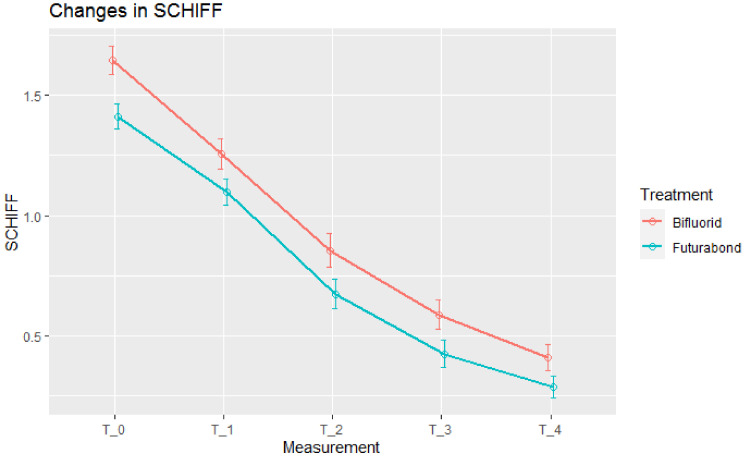
Pain intensity, Schiff measurements. Means ± std. errors.

**Figure 2:**
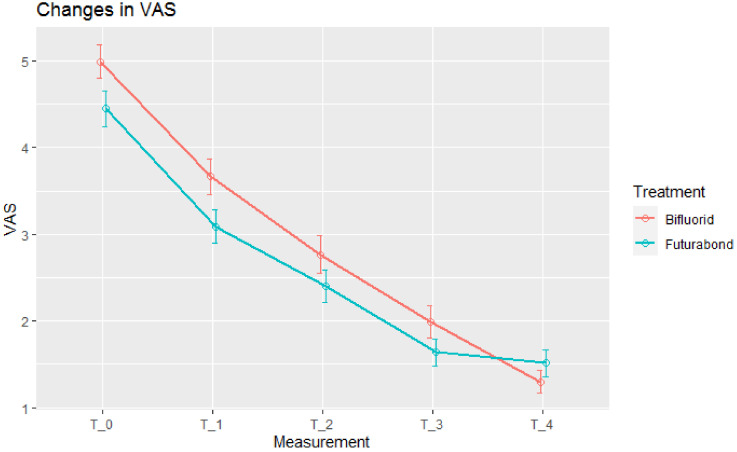
Pain intensity, Visual Analog Scale (VAS) measurements. Means ± std. errors.

There were 180 teeth treated by Bifluorid 10 and 160 by Futurabond U. At baseline, pain was slightly stronger in teeth treated with Bifluorid 10. Both treatments in both scales (SCHIFF and VAS) reduced pain intensity at each stage (Figure 1 and Figure 2).

### 3.2. The Effects of Bifluorid 10 and Futurabond U Expressed on SCHIFF Scale

Mean Schiff-measured pain reduction was statistically significant at each stage in both groups. At three of the four stages, total pain reduction was larger in Bifluorid 10 group, but the difference was not significant (Table 1).

### 3.3. The Effects of Bifluorid 10 and Futurabond U Expressed on VAS Scale

Mean VAS-measured pain reduction was statistically significant at each stage in Bifluorid 10 group and at three initial stages in Futurabond U group. At the fourth stage (T_3_–T_4_) pain reduction in Futurabond U group was very small and, at this stage, VAS-measured pain intensity became lower in Bifluorid 10 group (Figure 2). This made Bifluorid 10 treatment more efficient at T_3_–T_4_ stage as well as during all the observation period T_0_–T_4_ (Table 2).

### 3.4. Factors Influencing the Severity of Dental Hypersensitivity and Impact of Bifluorid 10 and Futurabond on Its Reduction

Regression models were used to estimate the influence of patient age and gender and BEWE on total pain reduction in both groups and according to both scales (Table 3). All the models were significant. Only patient age appeared to have significant influence. This influence is negative; pain in older patients reduced less than in younger (e.g., estimate of −0.013 for Schiff measurements, Bifluorid 10 group suggests, that expected pain reduction is 0.13 less for every 10 years of patients age).

## 4. Discussion

The present randomized controlled clinical split mouth study aimed to assess the long-term effectiveness of two in-office desensitizers: Bifluorid 10 and Futurabond U. The results showed that both the varnish and the bonding resin effectively reduced pain related to dentine hypersensitivity and that Bifluorid 10 was even more effective when pain reduction was assessed on VAS.

The etiology of dentine hypersensitivity based on the Brannstrom’s mechanism suggests that the best way to alleviate stimuli is to use a substance which blocks the contact between the tubules and external stimuli. The active agents should be able to be precipitated with tubules and occlude the dentin canals for a longer period of time. Such a protective surface was found in the studies in which fluoride [18], resins and adhesives [19] were active agents. In addition, glutaraldehyde is considered an effective active agent. However, it reacts with the tissues of the tooth in a different way to the previously mentioned agents. Glutaraldehyde and its derivates react with albumins in tubule fluid, leading to precipitate formation within tubules and subsequent narrowing or blocking of the tubules. However, glutaraldehyde is no longer used because of its side effects and low biocompatibility [20].

The current study enrolled 28 subjects, that received both the treatments, with a split-mouth design with randomized allocation. This study design allows to eliminate the bias related to interindividual variability. Bifluorid 10 was used for many years against dental hypersensitivity and is primarily dedicated by the producer to act against dental hypersensitivity [21]. In a previous study, it presented with an even better outcome than fluoride control [22]. On the other hand, Futurabond U is a dental adhesive for use alongside a methacrylate-based restorative, core build-up for luting materials and treatment of hypersensitive tooth necks. Dentin-bonding products, although not originally invented to treat hypersensitivity, have proved to be effective in reducing pain [18]. An additional advantage of Futurabond U system is the fact that it is mild self-etching (pH = 2.3) and is thus not so aggressive towards the smear layer (worth keeping in order not to expose the tubules and risk stronger pain stimuli).

The effectiveness of both groups of desensitizers is limited to their duration of remaining on tooth surface [22,23], which is difficult to exactly evaluate. Many factors could affect the duration of the desensitizing effect over time, including domestic oral hygiene procedures and desensitizers’ method of application and patients’ diet [24]. West et al., in a recent meta-analysis, highlighted a lack of studies with proper heterogeneity and controls [23,25]. In this clinical scenario, the results of this split-mouth study result in some interesting and reliable evidence. As a result, despite the noticeable pain reduction effect, no clear relationships can be drawn between personal factors, the duration of therapy or the type of agent used [16,23,25]. In a study by Duran et al., it was found that fluoride varnishes showed a worse outcome than bonding agents, contrary to the results of the present study [26]. A cross-sectional study by West et al. suggested that tooth wear due to erosion had a significant impact on the occurrence of dental hypersensitivity [27]. However, the present study shows that patient gender and BEWE of the tooth are insignificant regarding the therapeutic effect of both substances used. Although with age, the dentinal tubules undergo slow occlusion, we found that patient age has a significant negative influence on pain reduction. This indicates the need to deepen the differential diagnosis in order to find additional or even different etiology of pain [28]. Another interesting aspect highlighted by this study is associated with Schiff scale. In clinical studies on dentine hypersensitivity, this scale is intended to be sensitive only at the level of ≥2 [29] in order to reduce risk of bias. However, the same author included subjects with ≥1 and defined them as hypersensitive [30]. It is worth noting that the Schiff scale, although designed for the assessment of hypersensitivity, presents a narrow numerical range. This can be seen in the results of present research, where Bifluorid 10 and Futurabond U showed similar efficacy in reducing Schiff-measured pain reduction, while it was shown that Bifluorid 10 is significantly more efficient for VAS-measured pain reduction. The long follow-up period (6 months) makes it possible to avoid doubts that could arise from the fact that Bifluorid 10 was administered three times and Futurabond U once. The main differences found in VAS-scale pain were found between T3 and T4. The findings of this study are contrary to the recommendations of Hack et al., who suggest using varnishes only as for short-term management of dental hypersensitivity [31]. Moreover, clinical trials are being published in order to assess the clinical effectiveness and safety of novel formulations and coating agents in patients presenting different clinical conditions [32,33,34].

## 5. Conclusions

1. Bifluorid 10 and Futurabond U are effective in the treatment of dental hypersensitivity.
2. Bifluorid 10 and Futurabond U have similar efficacy in reducing SCHIFF-measured pain reduction, while Bifluorid 10 is significantly more efficient for VAS-measured pain reduction, mainly due to reduction at last stage of study (i.e., 2–6 month after last treatment).
3. Patient’s age has a significant negative influence on pain reduction (both SCHIFF and VAS-measured), while influence of patient’s gender and BEWE of the tooth is insignificant.

## Figures and Tables

**Table 1:** Significance of pain reduction at four stages and total pain reduction in Bifluorid 10 and Futurabond U groups and difference between groups accordingly to Schiff measurements. Positive value of t-statistic in “Difference” row indicates larger efficacy of Bifluorid 10, negative values of Futurabond U.

		Pain Reduction by Stage				Total Pain Reduction (T_0_–T_4_)
		T_0_–T_1_	T_1_–T_2_	T_2_–T_3_	T_3_–T_4_	
Bifluorid 10	Mean	0.389	0.400	0.267	0.178	1.233
	t-statistic	8.81	9.66	5.25	4.33	24.72
	*p*-value	<0.001 *	<0.001 *	<0.001 *	<0.001 *	<0.001 *
Futurabond U	Mean	0.313	0.425	0.250	0.138	1.125
	t-statistic	7.66	9.88	5.43	3.35	21.52
	*p*-value	<0.001 *	<0.001 *	<0.001 *	0.001 *	<0.001 *
Difference	t-statistic	1.27	−0.42	0.24	0.69	1.50
	*p*-value	0.205	0.676	0.808	0.489	0.135

*—characteristic significant at 0.05 level.

**Table 2:** Significance of pain reduction at four stages and total pain reduction in Bifluorid 10 and Futurabond U groups and difference between groups according to VAS. Positive value of t-statistic in “Difference” row indicates a higher efficacy of Bifluorid 10, negative values of Futurabond U.

		Pain Reduction By Stage				Total Pain Reduction (T_0_–T_4_)
		T_0_–T_1_	T_1_–T_2_	T_2_–T_3_	T_3_–T_4_	
Bifluorid 10	Mean	1.322	0.900	0.778	0.689	3.689
	t-statistic	10.54	9.39	7.29	4.47	19.73
	*p*-value	<0.001 *	<0.001 *	<0.001 *	<0.001 *	<0.001 *
Futurabond U	Mean	1.363	0.688	0.763	0.125	2.938
	t-statistic	8.81	6.99	7.20	1.13	16.31
	*p*	<0.001 *	<0.001 *	<0.001 *	0.259	<0.001 *
Difference	t-statistic	−0.20	1.55	0.10	2.97	2.89
	*p*-value	0.840	0.123	0.919	0.003*	0.004 *

*—characteristic significant at 0.05 level.

**Table 3:** Regression analysis of total pain reduction in both groups according to SCHIFF and VAS measurements.

**SCHIFF, Bifluorid 10**				
**Variable**	**Estimate**	**Std. Error**	**t-Statistic**	***p*-Value**
(Intercept)	1.689	0.221	7.644	<0.001 *
Age	−0.0130	0.0042	−3.118	0.002 *
GenderM	−0.155	0.118	−1.310	0.192
BEWE	0.1126	0.0598	1.882	0.061
Model characteristics	R^2^ = 0.077 F (3, 176) = 4.868; *p* = 0.002			
**SCHIFF, Futurabond U**				
**Variable**	**Estimate**	**Std. Error**	**t-Statistic**	***p*-Value**
(Intercept)	1.704	0.207	8.231	<0.001 *
Age	−0.0127	0.0040	−3.208	0.002 *
GenderM	−0.181	0.119	−1.529	0.128
BEWE	0.0257	0.0636	0.405	0.686
Model characteristics	R^2^ = 0.067 F(3, 156) = 3.76; *p* < 0.012			
**VAS, Bifluorid 10**				
**Variable**	**Estimate**	**Std. Error**	**t-Statistic**	***p*-Value**
(Intercept)	7.314	0.810	9.021	<0.001 *
Age	−0.0688	0.0153	−4.481	<0.001 *
GenderM	0.207	0.434	0.477	0.634
BEWE	−0.4040	0.220	−1.839	0.068
Model characteristics	R^2^ = 0.114 F(3, 176) = 7.56; *p* < 0.001			
**VAS, Futurabond U**				
**Variable**	**Estimate**	**Std. Error**	***t*-Statistic**	***p*-Value**
(Intercept)	4.859	0.698	6.965	<0.001 *
Age	−0.0492	0.0133	−3.696	<0.001 *
GenderM	0.6011	0.400	1.503	0.135
BEWE	0.0770	0.214	0.359	0.720
Model characteristics	R^2^ = 0.108 F(3, 156) = 6.267; *p* < 0.001			

*—characteristic significant at 0.05 level.

## Data Availability

Data are available upon request.
